# Elevational is the main factor controlling the soil microbial community structure in alpine tundra of the Changbai Mountain

**DOI:** 10.1038/s41598-020-69441-w

**Published:** 2020-07-24

**Authors:** Mingze Tang, Lin Li, Xiaolong Wang, Jian You, Jiangnan Li, Xia Chen

**Affiliations:** 1 National & Local United Engineering Laboratory for Chinese Herbal Medicine Breeding and Cultivation, School of Life Sciences, Jilin University, Changchun, 130112 China; 2 Medical Technology Department, Qiqihar Medical University, Qiqihar, 161006 China

**Keywords:** Ecology, Ecology, Environmental sciences

## Abstract

To reveal the self-coordination mechanism of the fragile ecosystem of alpine tundra, we explored the relationship between soil microorganisms and other elements. On the alpine tundra of the Changbai Mountain, different vegetation types, altitudes and soil properties were selected as driving factors of soil microbial community. Soil microbial community, C- and N-cycling functional microbial and fungal biomass were analyzed. Structural equation model was used to study the control of biotic and abiotic factors in rhizosphere soil microbial community. The results showed that the pH value of soil had the strongest direct impact on the diversity and community structure of soil microorganisms, and had significant correlation with most of the C- and N-cycling functional microbial; organic carbon and vegetation also have strongest direct effect on fungal biomass, but all of them were not main factors influence soil microbial community structure, the elevation was the main controlling factor. In addition, the elevation mainly through indirect action affects the soil microbial community by driving distribution of plant species, soil organic carbon and pH value. This finding highlighted that elevation was the main predictor to determine rhizosphere microbial community structure but not vegetation in alpine tundra of Changbai Mountain.

## Introduction

Soil microorganism as one of the most important links in the biochemical cycle, can affect many important cycling processes of soil ecosystem^1^, such as carbon and nitrogen cycling in the ecosystem, transformation and cycling of soil nutrients (nitrogen, phosphorus, etc.). Therefore, changes in soil microbial community structure are often used as important sensitive indicators of changes in soil environment and nutrients^2^. Given that the influence of environmental factors on microorganisms can reveal fundamental problems in microbial ecology^3,4^, research on it will be of great significance for the function of soil ecosystem^5^.

Vegetation plays an important role in shaping the rhizosphere microbiome^6,7^. It has been found that narrow areas of soil around plant roots are affected by root activity and/or root exudates^8^, and that each vegetation type supports a rhizosphere-specific microbial community^9,10^. Thus, specific soil conditions under different vegetation types coexist with specific soil microbial communities^11^. As a frontier ecological topic in the field of microbial–environmental interaction^12,13^, a deeper understanding of the relationship between vegetation types and soil microbial communities may help us develop better strategies to protect the harsh environment of alpine tundra^14^.

Alpine tundra elevation gradients have also proven to be important when people try to understand the effects of environmental factors on the structure and distribution of microbial communities. A short elevation gradient on a high mountain can provide a serious environmental gradient. Therefore, the species number and richness of which decrease with the elevation rising^15^, and the composition and metabolic rate of soil microbial communities can also be regulated^16^. To date, some is known regarding the shift in microbial community structure and functional genes along an alpine climosequence^17–19^. Although the assessment of effects such as high turnover of aboveground vegetation, local soil conditions, and climate regimes on spatial patterns of microbial communities along elevation gradients^20^ has been performed, most experimental studies have focused on large-scale natural ecosystems or microbial communities along latitudinal gradients^21,22^ rather than across the range and scale of elevation gradients^23,24^.

Besides vegetation and elevation gradient, soil microbial is also sensitive to soil properties such as pH, moisture, nutrient amounts, and nitrogen/carbon (C/N) ratio^25–27^. Uroz et al. observed that bacterial community composition was strongly correlated with soil characteristics under different vegetation types^27^. Similarly, Shi et al. have recently observed that bacterial, fungal, and eukaryotic communities were correlated with ammonium concentration, dissolved organic nitrogen (DON) content, and C/N ratio in the Arctic tundra^28^. In addition, as important component of the soil microbial community, some studies have suggested that the biomass of fungi is significantly correlated with soil nutrients, because of relatively high amounts of chitin and protein in fungal cell walls and their biopolymers, thus representing an important source of both carbon (C) and nitrogen (N)^29–31^. The relationships between soil physicochemical characteristics and the development of plant and microbial successions have been subject of investigation for a long time^27^.

The above relationships are all interrelated. Vegetation along the mountainside elevation distribution may vary, when mirroring the latitudinal vegetation gradient from temperate to frigid zones on the Eurasian continent^32,33^. Soil properties such as pH and nutrient availability are also important drivers of structure, diversity, and functioning of both plant and microbial communities^34–36^. However, little studies taking all of them into account.

The objectives of this study were (1) to explore the relationship among vegetation, elevation, soil physicochemical properties and soil microbial communities, (2) to determine the main driving factor controlling the microbial community. Considering that climate change and the change of vegetation type which caused by alien species invasion can pose an important challenge to the environmental protection of tundra, our study should provide scientific basis for predicting the change trend of microbial community under different environmental conditions. Moreover, in view of the important position of soil microorganisms in the terrestrial ecosystem, to explore the relationship between soil microorganisms and other elements of the tundra ecosystem will help us to understand the self-coordination mechanism of the whole ecosystem and provide theoretical basis for environmental protection or disaster recovery.

In this study, the soil bacterial communities in the alpine tundra of Changbai Mountain were studied at different elevations (2,000–2,600 m) and under different conditions of vegetation rhizospheres and soil environments. Due to the plant rhizosphere soil as our research object which is specifically influenced by plant root activities and/or root exudates, we further hypothesized that vegetation may be the main controlling factors for microbial community composition in rhizosphere soil. Therefore, we set up two research groups along the elevation gradient to study the relationship between soil biological factors, abiotic factors and microbial communities under different dominant vegetation types and different mixed types of the same species (see “Experimental design” method). We used the sequencing method (Illumina MiSeq) to analyze the soil microbial community structure, real-time PCR to quantitatively analyze the nutrient cycling functional genes, and high-performance liquid chromatography (HPLC) to quantify the fungal biomass, and related it to the elevation, species investigation and measurement of soil variables to reveal the potential interaction.

## Results

### Rhizosphere soil bacterial community along the elevation gradient

**Figure 1:**
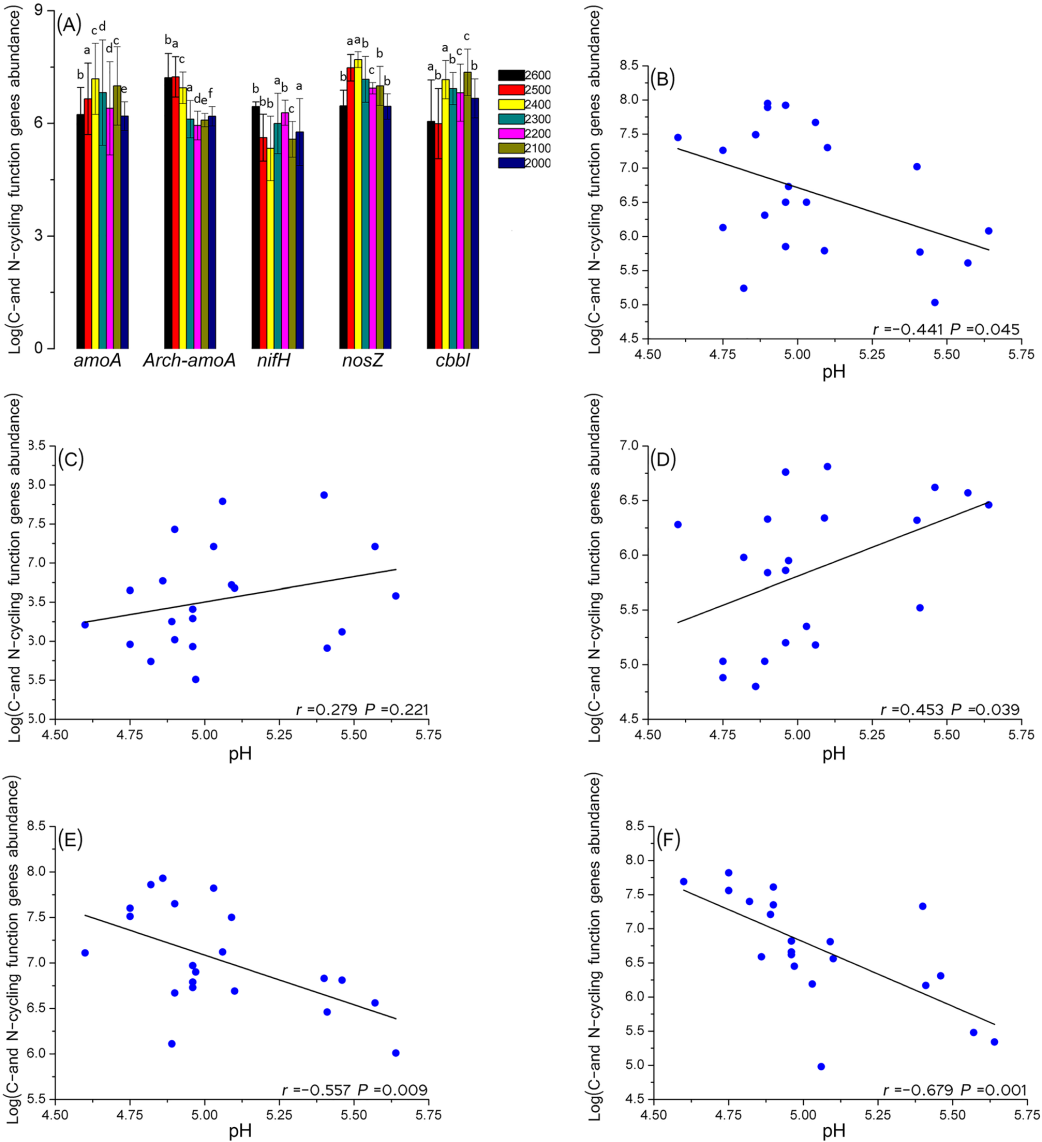
(**A**) Real-time PCR quantification of the C- and N-cycling function genes *(aomA* bacterial *amoA*, *Arch-amoA* archaeal *amoA*, *nifH* nitrogen-fixing bacteria functional gene, *nosZ* denitrifying bacteria functional gene, *cbbl* carbon-fixing bacteria functional gene) in the bulk soil under seven elevations. The copy number of genes in each gram of dry soil was estimated based on the results of real-time PCR (copies per ng DNA). The standard curve of all these genes was > 0.99. Each sample was measured in triplicate. (**B**–**F**) Pearson correlations between C- and N-cycling function genes abundance and pH-value. (**B**) was bacterial *amoA*; (**C**) was archaeal *amoA*; (**D**) was *nifH*; (**E**) was *nosZ* and (**F**) was *cbbl* correlation with pH. Pearson correlations between C- and N-cycling function genes abundance and other indexes see Supplementary Table S2.

Soil nutrients significantly varied across the elevation gradient (Supplementary Table S1); soil enzyme activity did not significantly vary, with the exception of urease activity. Ergosterol levels were the highest at the elevation 2,200 m and the lowest at 2,600 m (Supplementary Fig. S1), but fungal biomass did not differ significantly (*P* = 0.079). Correlation analyses (Supplementary Fig. S2) showed that fungal biomass was significantly positively correlated with TN (total nitrogen), TOC (total organic carbon), MBC (microbial biomass carbon), and sucrase (P ≤ 0.001). Assessment of the abundance of C- and N-cycling functional groups (bacterial *aomA* ammonia oxidizing bacteria functional gene, archaeal *aomA* ammonia oxidizing archaea functional gene, *nifH* nitrogen-fixing bacteria functional gene, *nosZ* denitrifying bacteria functional gene, *cbbl* carbon-fixing bacteria functional gene) revealed that only the abundances of archaeal *aomA* and *nosZ* significantly differed (*P* ≤ 0.05) among the seven elevations (Fig. 1A). Pearson´s correlation coefficient (r) for the abundances of C- and N-cycling function genes and soil properties showed that the functional genes were significantly (*P* ≤ 0.05) correlated with pH values, except for archaeal *aomA* genes (Fig. 1B–F). Among them, the abundance of archaeal *aomA* was correlated with elevation, soil moisture, and urease, the abundance of *cbbl* was positively correlated with SOC, C/N ratio, urease, and soil moisture and negatively correlated with NH_4_^+^. The abundance of *nosZ* was positively correlated with phosphatase and bacterial *aomA* was positively correlated with soil moisture (Supplementary Table S2).

16S rRNA gene sequencing showed that alpha diversity was significantly different between the seven elevations, except in terms of the Simpson index (Table 1). The OTU richness exhibited a unimodal pattern with elevation, albeit with no significant differences (*P* = 0.255), and showed higher values for samples from lower or higher elevations than for samples from medium elevations (Supplementary Fig. S3).

**Table 1:** The alpha diversity difference between seven elevation treatments.

	Chao1	Observed species	PD whole_tree	Shannon	Simpson
2,600 m	1,207.291	1,049.333	60.10461	8.382897	0.991891
2,500 m	896.4872	746.3333	45.823	7.435108	0.987337
2,400 m	1,089.604	902.3333	55.01141	7.662607	0.987238
2,300 m	913.1888	757	46.78453	7.431501	0.985501
2,200 m	1,240.182	988	57.33918	7.842169	0.985375
2,100 m	1,017.882	830.3333	51.05846	7.643039	0.988449
2000 m	1,118.46	908.3333	52.47835	7.637198	0.984229
*P*-value	**0.025**	**0.008**	**0.031**	**0.005**	0.173

Bolded values indicate significant (*P* < 0.05, ANOVA) effects.

**Figure 2:**
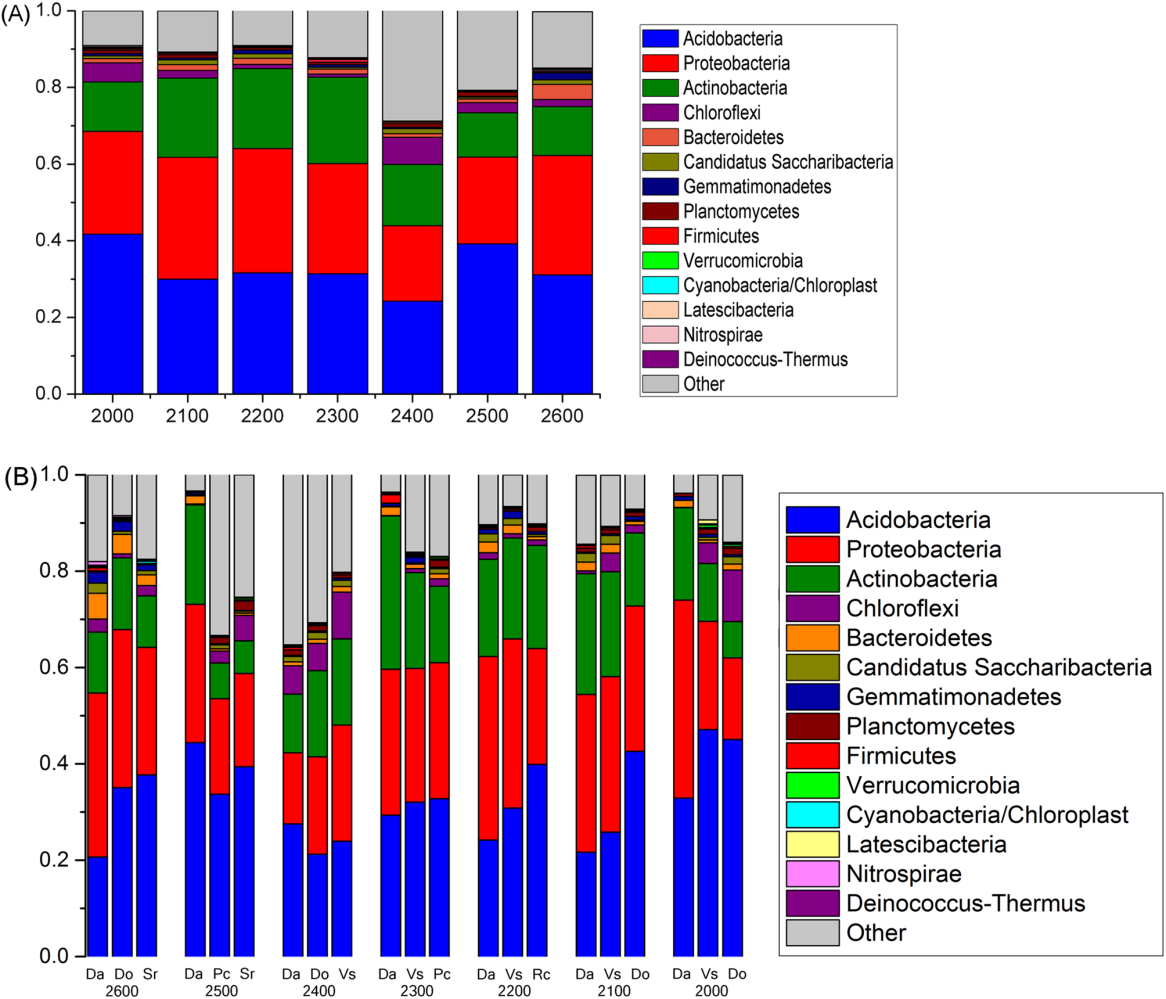
Relative abundances of the dominant bacterial phyla in soils separated according to elevation and species categories. Relative abundances are based on the proportional frequencies of those DNA sequences that could be classified at the phylum level. (**A**) was separated according to elevation categories; (**B**) was separated according to species categories at seven elevations.

**Figure 3:**
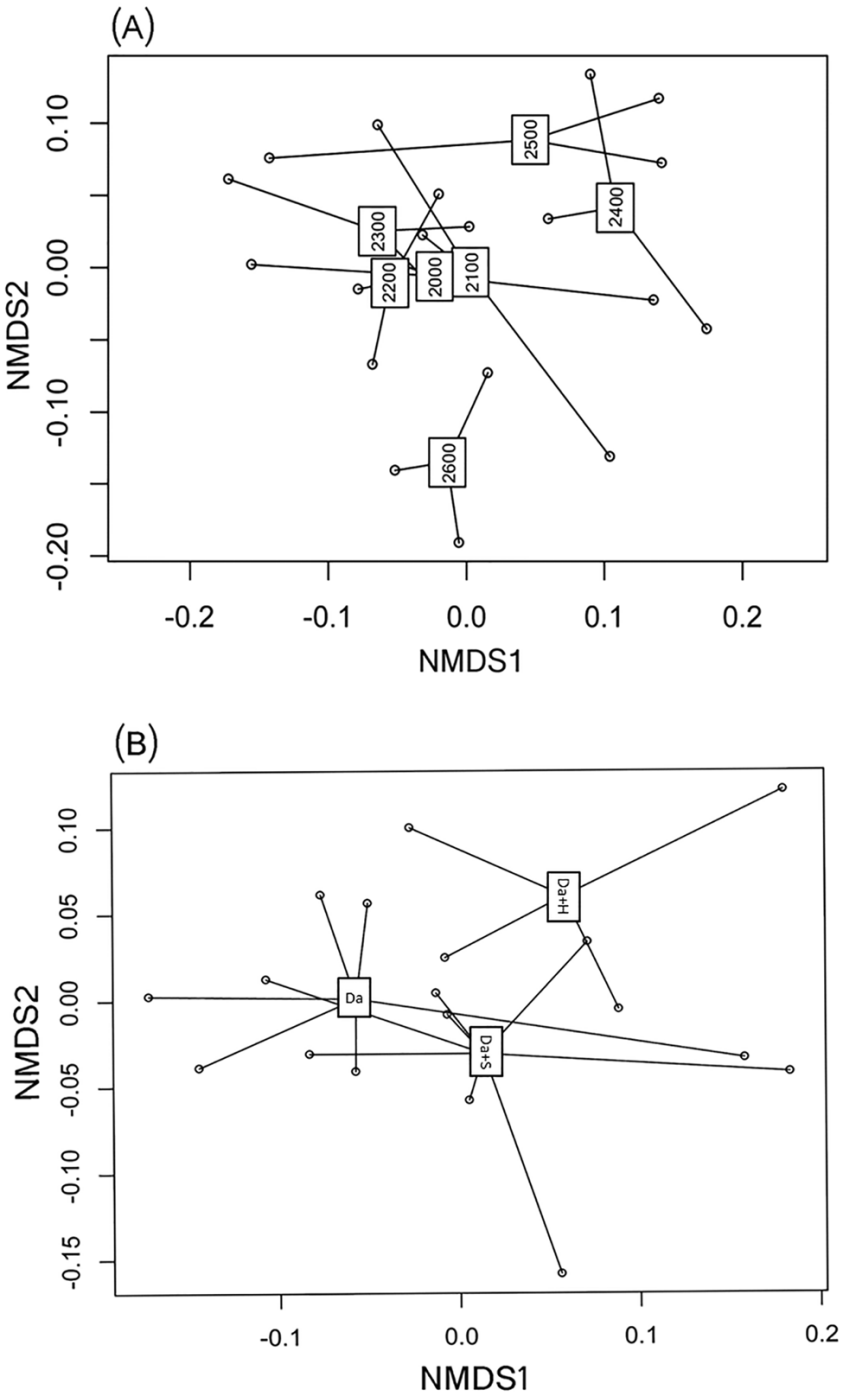
Non-metric multidimensional scaling (NMDS) plot of community composition based on pyrosequencing of (**A**) microbial communities of seven elevations and (**B**) microbial communities of Da (the independent community of *R. aureum Georgi*), Da + S (*R. aureum Georgi* lives with other shrub), Da + H (*R. aureum Georgi* lives with herbaceous). Distances for (**A**,**B**) are based on weighted Unifrac scores. Bray-Stress for (**A**,**B**) are 0.134 and 0.082.

In terms of the response of the bacterial community to the different elevations, Acidobacteria (33%) was the most dominant phylum, while Proteobacteria was the second most abundant phylum, accounting for 28% of all sequences; Actinobacteria were accounting for 17% (Fig. 2A). Testing by ANOSIM revealed that three dominant bacterial phyla (Bacteroidetes, Gemmatimonadetes, and Nitrospirae) showed significantly different abundances between different elevations (Supplementary Table S3). Nitrospirae was most abundant at 2,600 m, but non-existent at elevations of 2,500, 2,400, and 2,100. Non-metric multidimensional scaling (NMDS) ordination revealed microbial communities were significantly different (*P* = 0.011) between elevations (Fig. 3A). The microbial community at 2,600 m was separate from that of other elevations, but communities at 2,500 and 2,400 m were more similar. The microbial community at lower elevations (below 2,300 m) was not obviously different (Fig. 3A).

### Different rhizosphere soil bacterial communities at similar elevations

**Figure 4:**
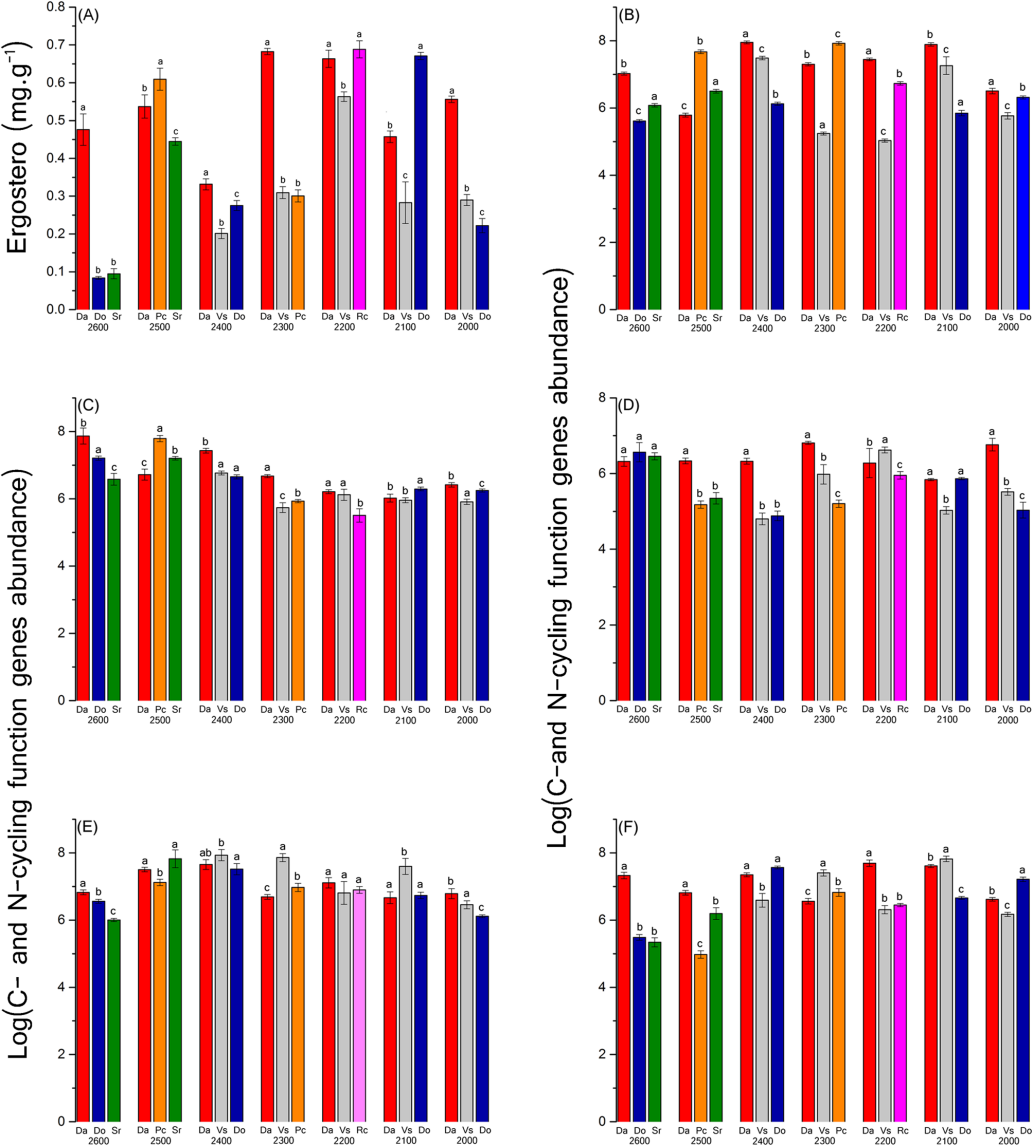
(**A**) Ergosterol (mg g^−1^) in rhizosphere soil for different species on the seven elevations. (**B**–**F**) Real-time PCR quantification of the C- and N-cycling function genes in the rhizosphere soil under different species on the seven elevations. (**B**) is bacterial *amoA*, (**C**) is archaeal *amoA*; (**C**) is *nifH*; (**D**) is *nosZ* and (**F**) is *cbbl*.

The results of the ANOVA showed the rhizosphere soil properties of different plants at the same altitude were different significantly. At 2,600, 2,500, 2,200, and 2,000 m, total nutrient levels at Da (the independent community of *R. aureum Georgi*) sites were higher than at other sites. However, soil pH was not significantly different among the three plant soils at same elevations of 2,600, 2,500, and 2,100 m. Soil enzymatic activities at Da sites were highest at 2,600 and 2,100 m (Supplementary Table S1). At the same elevations, the content of ergosterol was significantly different between the three plant soil samples (Supplementary Table S7). Among them, Pc, Rc, and Do had the highest ergosterol contents, respectively, at 2,500, 2,200, and 2,100 m; ergosterol contents of Da sites were higher than others at the remaining three elevations (Fig. 4A).

Abundance of C- and N-cycling functional groups in the rhizosphere soil, for bacterial *amoA*, was highest at 2,500 and 2,100 for Pc. At other elevations, highest abundance was found for Da sites. The abundance of archaeal *amoA* (Fig. 4B–F) was highest in Pc and Do sites at 2,500 and 2,300 m respectively; Da sites showed higher values than the two other sites at the remaining elevations. For *nifH* abundance, there was no significant difference among the three plant species at 2,600 m; Vs had the highest abundance at 2,200 m, at other three elevations, the highest abundance of *nifH* was found in Da sites. For nosZ abundance, values at 2,200 m were not significantly different between plant species. The rhizosphere soil of Da had the highest abundance of *cbbl* at elevations of 2,600, 2,500 and 2,200 m.

**Figure 5:**
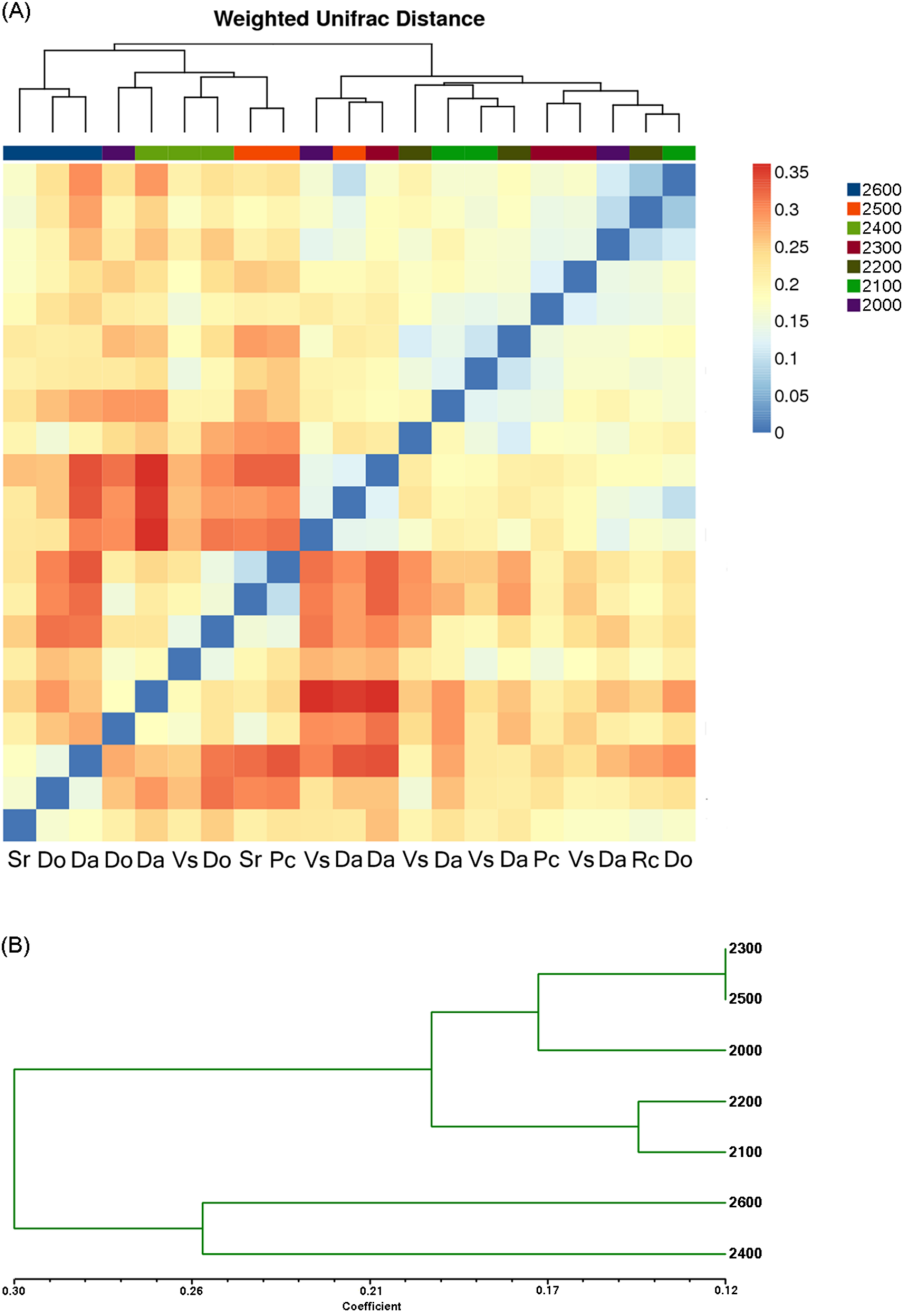
(**A**) Pairwise comparison of β-diversity with all samples and annotations. Clustering and heatmap were computed using the weighted Unifrac scores. (**B**) Hierarchical clustering analysis of microbial communities for Da at seven elevations based on pyrosequencing data.

Taxonomic summary of the relative abundance of bacteria (Fig. 2B) showed that rhizosphere soil microbial communities were different between the three plant species at the same elevation. The rhizosphere soil of Da had a higher relative abundance of Proteobacteria (phyla) than other plant species at the same elevation, except at 2,400 m. The heatmap shows pairwise comparisons of the bacterial community structures between all samples (Fig. 5A) showed that the sites at 2,600 m were clustered into one clade. At 2,500, 2,400, and 2,300 m, the Da sites were separated from the other two sampling sites. Soil communities were clustered into different clades at lower altitudes. Hierarchical clustering analysis (Fig. 5B) for Da at seven elevations showed that 2,600 m and 2,400 m were clustered into one clade, Da at other elevations formed clustered into the other clade.

### The rhizosphere soil bacterial community in different plant communities formed by the same vegetation

The rhizosphere soil properties of different plant communities formed by the same plant in the alpine tundra were not significantly different (Supplementary Table S4). The highest content of ergosterol in the soil was found for Da; Da + S (*R. aureum Georgi* lives with other shrub) had lower content and Da + H (*R. aureum Georgi* lives with herbaceous) the lowest (Supplementary Fig. S4).

**Figure 6:**
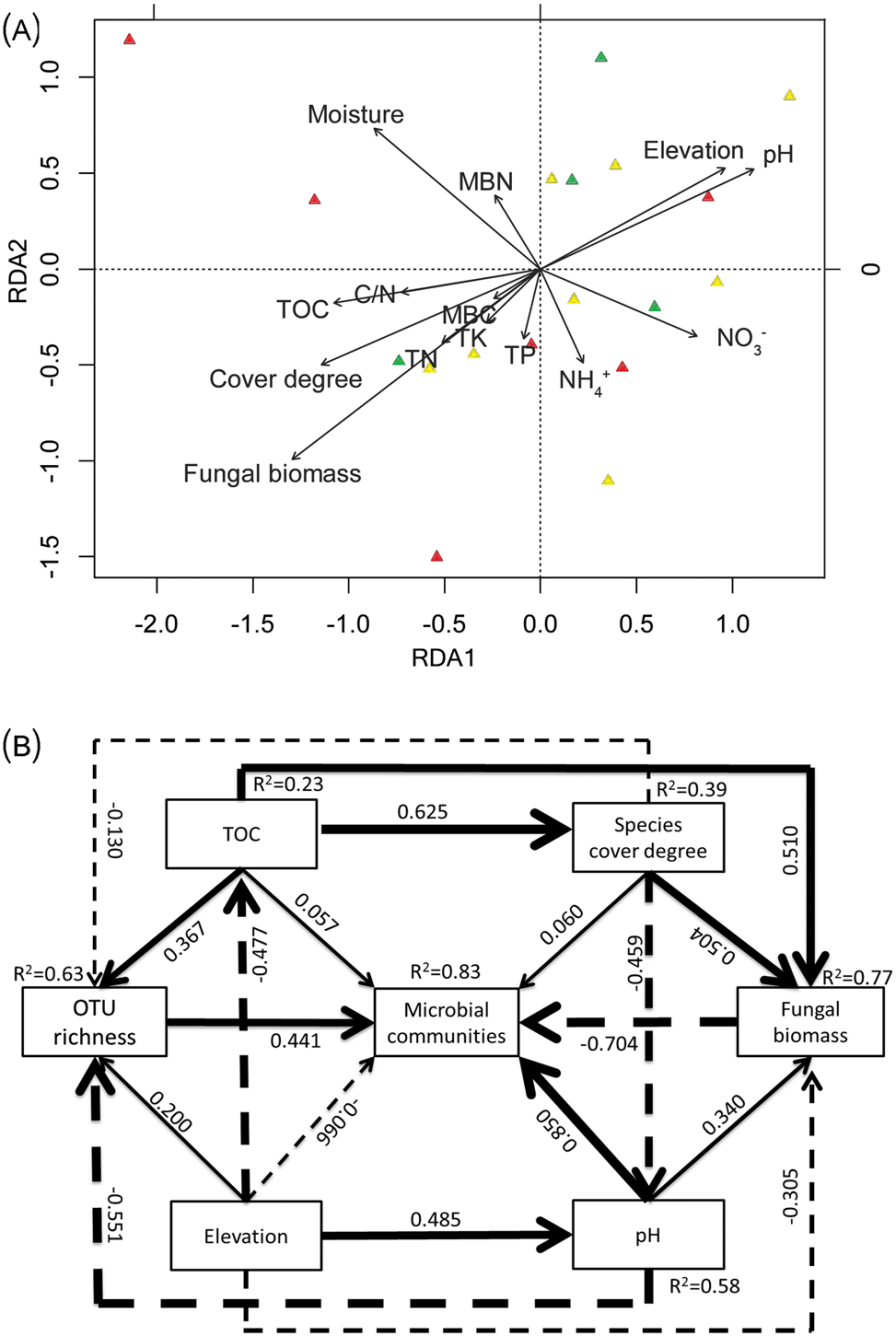
(**A**) Redundancy analysis (RDA) triplots of 16S rDNA fingerprint patterns, showing the contribution of 14 environmental parameters to variability. Arrows indicate environmental factors and their relative effects on microbial community structure. The red triangles indicate Da; yellow triangles indicate Da + S; green triangles indicate Da + H. Eigenvalues of RDA1 and RDA2 are 0.336 and 0.2436. (**B**) A structural equation model (SEM) showing the causal influences of soil TOC, pH, species cover degree, elevation, OTU richness, fungal biomass and microbial communities in the soil. The width of arrows indicates the strength of the causal effect. The numbers above the arrows indicate path coefficients (*indicate significant (*P* < 0.05) effects, **indicate significant (*P* < 0.01) effects, ***indicate significant (*P* < 0.001) effects). Bold and dashed lines indicate positive and negative effects respectively. *R*^2^ values represent the proportion of the variance explained for each variable.

The abundance of C- and N-cycling functional groups was assessed for three treatments. Treatment Da + H had the lowest abundance of nifH and *cbbl* genes, but the abundance of other functional genes was not significantly different between the three treatments (Supplementary Fig. S5). There was no significant difference in OTU richness among the three plant communities (Supplementary Fig. S6). Pearson correlation analysis indicated that OTU richness and fugal biomass were significantly intercorrelated and both were significant positively correlated with TOC and species degree (*P* < 0.05), but negative correlated with pH (*P* < 0.05) (Supplementary Table S5). Fugal biomass was also negatively correlated with elevation (*P* < 0.05). NMDS ordination revealed that there was no significant difference (*P* = 0.145) in microbial communities among Da, Da + S and Da + H (Fig. 3B). However, microbial communities at Da + S and Da + H sites were more similar than at Da. In the redundancy analysis (RDA) ordination biplot (Fig. 6A), fungal biomass and cover degree showed a significant correlation with community composition (Supplementary Table S6). Other factors, such as moisture, pH, elevation, and TOC also showed a high correlation with bacterial community composition (Supplementary Table S6).

In order to further analyze the relationship between soil biological factors and non-biological factors, the structural equation model (SEM) was constructed. The path model on the controls of soil microbial community structure by dominant site factors passed all the statistical tests on adequacy (λ^2^ = 0.980, *P* = 0.806, CMIN /df = 0.327; GFI = 0.990; CFI = 1.000; RMSEA < 0.001) and explained 63%, 83%, and 77%, respectively, of the variance in the abundance of OTU richness, bacterial communities and fungal biomass. The path coefficients (λ) for direct effects are displayed in Fig. 6B. The direct, indirect, and total effects on bacterial communities and intermediate explanatory variables are shown in Table 2. Regarding the total effects, elevation (λ = 0.603) was the strongest predictor for microbial community composition in rhizosphere soil compared TOC, pH and vegetation coverage (Table 2). Regarding the direct effects, pH (λ = 0.850, *P* < 0.001) was the strongest direct predictor for microbial communities in rhizosphere soil, followed by fungal biomass (λ = − 0.704, *P* < 0.001) and OTU richness (λ = 0.441, *P* = 0.008). The direct effects of TOC (λ = 0.057, *P* > 0.05), elevation (λ = − 0.066, *P* > 0.05), and vegetation cover degree (λ = 0.060, *P* > 0.05) were relatively weak (Fig. 6B); these factors only influenced microbial communities mainly through the indirect path. Elevation was the strongest indirect predictor (mediated through pH, TOC, fungal biomass, and OTU richness), followed by pH (mediated through OTU richness and fungal biomass), vegetation cover degree (mediated through fungal biomass, pH, and OTU richness), and TOC (mediated through cover degree, fungal biomass, and OTU richness), whereas the effect of elevation, pH, vegetation cover degree, and TOC on bacterial communities was through a combination of both direct and indirect paths. Among them, soil TOC and the vegetation cover degree for fungal biomass had the largest direct effect (λ = 0.504, *P* < 0.05) of all parameters. Soil pH had a positive direct effect on microbial community structure, but a negative indirect one.

**Table 2:**
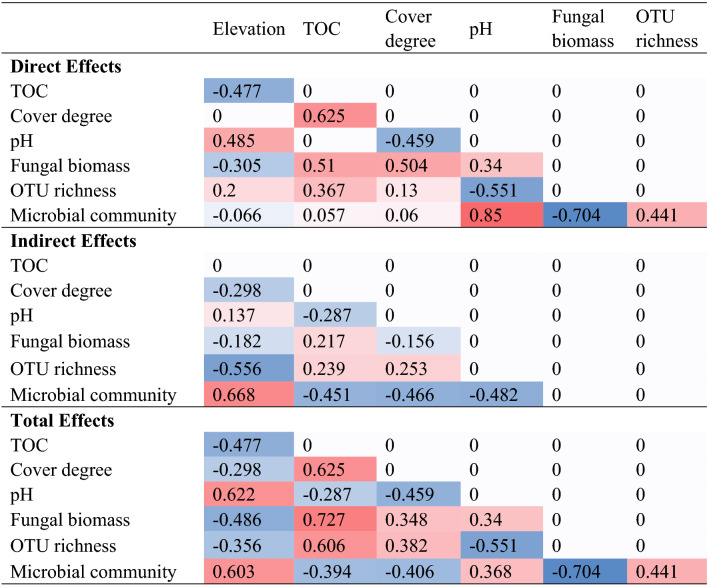
Direct, indirect and total effect coefficients of each variable.

Stronger colours (red is positive, blue is negative) in the heatmap represent stronger effect.

## Discussion

### Effects of vegetation on the microbial community

The interaction between plants and soil microbial is one of the forefront topics of international ecological research^12,13^. In our research, soil nutrients, fungal biomass, and nutrient cycling gene abundance of different plant rhizosphere soils were significantly different at similar altitudes. Host specificity to a species or group of related species is also well documented in plant-associated fungi^37,38^. Some studies have shown strong impacts of vegetation on the abundance of C- and N-cycling soil bacteria and archaea^39^. In addition, our result showed that N-fixing gene (*nifH*) and C-fixing gene (*cbbl*) abundance were significant different. Aureum plant community lives with other species. Further said that the variation of species have an impact on functional microbial abundance.

In-depth analysis showed that *R. aureum Georgi* rhizosphere soil had higher soil nutrient, fungal biomass, and C- and N-fixing gene abundance at most elevations compared to other species, as well as higher nutrient cycling rates. Plants and their microbiomes can be considered as “superorganisms”, partly due to their reliance on soil microbiota for specific functions and traits^13,40^. Plant species appear to be an important factor for soil N transformation in the alpine tundra, and, consequently, may influence plant community structures and ecosystem functions^41,42^. This might be one of the reasons why *R. aureum* was abundant at each altitude.

The microbial community structure in different vegetation rhizosphere soils at similar elevations was different. This is in agreement with the findings of previous studies that different vegetation types significantly affect the microbial community structure and functional diversity in rhizosphere soil^43,44^. Moreover, NMDS ordination revealed that mixing with herbaceous species resulted in higher dissimilarly than mixing with shrub species. Different plant types have different effects on the soil^43,45^, and soil microbial community structure over altitudinal gradients is mainly governed by changes in vegetation cover type^46^.

### Effects of elevation gradients on the microbial community

Elevation strongly affects rhizosphere soil properties. The contents of TN, TOC, water content, C/N, urease decreased with the increase of elevation which is consistent with the previous research results^45^. There was no significant correlation between fungal biomass and elevation gradient. Similarly, previous studies have shown no significant difference in fungal biomass at different altitudes^38^. Our study found that with the decrease of altitude, the fungal biomass related to the same shrub root system increased. Margesin et al. also suggested that fungal biomass decreased with altitude. In the high altitude area, the environment condition is harsh, and the fungi associate with roots of the host plants, thereby increasing absorptive surface area and allowing enhanced uptake of essential nutrients and water^47^. Similarly, soil fungal biomass has been reported to vary along elevation gradients^48,49^. Although different fungal groups produce different levels of ergosterol (which may not exist in some fungal groups)^50,51^, few fungi (< 5% of OTUs) are found lacking ergosterol, and all communities have similar order-level composition^38^, which allows for comparison of biomass concentrations. Contrary to fungal biomass, C- and N-cycling gene abundances did not vary along elevation gradients, but there were significant differences in different vegetation types. Shen et al. suggest that soil microbial functional gene richness dramatically increases at the treeline ecotone from temperate forest to alpine tundra in the Changbai Mountains^52^. Different vegetation types with different litter input which can lead to difference in the composition of soil organic matter^53^. Tian et al. using the Biolog method found that labile organic carbon contribute to variation in functional microbial diversity^54^. This indicates that vegetation types drive the spatial distribution of functional gene richness along elevation gradients in alpine tundra. To the best of our knowledge, this finding has not been reported in a small-scale elevation gradient in alpine tundra studies.

Taxonomic richness does not follow the classic decreasing or unimodal elevation diversity patterns, which agrees with most studies finding non-significant elevation patterns^52,55,56^. Shen et al. observed that taxonomic richness linearly decreased with increased elevation and phylogenetic diversity exhibited a unimodal pattern with elevation^57^. This observation is not in agreement with our results, possibly because these authors were studying the bulk soil, while we focused on rhizosphere soil influenced by plant roots and a zone of high microbial activity, clearly distinct from bulk soil^58^. Here, we found similar bacterial communities at higher elevations (2,600, 2,500, and 2,400 m), but dissimilarities at lower elevations. Based on the results of the RDA, the bacterial community composition was significantly affected by altitude and three phyla (Bacteroidetes, Gemmatimonadetes, and Nitrospirae). Abundance of Nitrospirae, belonging to the nitrite-oxidizers, was highest at 2,600 m. Nitrifying bacteria appear significantly impacted by elevation and may be drivers of living at high altitude. Many researchers have found that bacterial or micro-eukaryotic composition varied with elevation^55,56,59,60^. As a consequence, elevation is a main factor contributing to the overall bacterial community, but different vegetation types also interfere with the composition of the rhizosphere soil microbial community structure.

### Effects of soil properties on the microbial community

There is accumulated evidence that soil physical and chemical properties are usually the best predictors of variations in microbial diversity^42,61,62^. Fungal biomass was significantly correlated with soil TOC, TN, MBC, and PH. José et al. stated that fungal abundance at higher altitudes was significantly and positively related to the high amounts of C (TOC and humus), EC, N, and mineral nutrients in subalpine and alpine environments^63^. The higher C, N, and P levels with increasing altitudes may contribute to enhanced microbial growth^63^. As previously observed, the bacterial diversity/structure and function was strongly influenced by soil properties^34,52,64,65^. In our research, correlation analyses demonstrated that C- and N-cycling gene (except ammonia-oxidizing archaea) abundance is associated with pH, some of them also correlated with soil moisture, TOC, C/N, and acid phosphatase. Previous studies have suggested that N-cycling is usually associated with NH_4_^+^ or NO_3_^−^, but we did not observe this in our study. This result was to be expected, since gene presence is not necessarily related to gene activity^66^. Similar studies have reported that N-cycling functional genes were contingent on local site characteristics such as soil nutrients, soil temperatures, and site latitude^66–68^.

Similar to N-cycling genes, soil pH also has a stronger influence on soil microbial community structure than some other parameters. This agrees with other studies which found that the dominant bacterial taxonomic diversity/structure was strongly influenced by soil pH^62,69^. Here, pH was significantly correlated with OTU richness, fungal biomass, and other soil properties, especially soil TOC, which strongly influenced microbial community.

In summary, our study reveals complex impacts of the microenvironment by various elevation, species cover degree, and soil properties and their interactive effects, using the SEM. Rhizosphere soil microbial community composition was most strongly influenced by fungal biomass, followed by elevation. Elevation, species cover degree, and soil TOC had little direct effect on the microbial composition, while TOC and species cover degree were most strongly affected by fungal biomass. Elevation also had a strong effect on fungal biomass. Previous studies have demonstrated no significant variation in fungal biomass between elevation zones; instead, ergosterol varied according to host species type^70^ and fungal richness was primarily affected by host species identity^71^. Moreover, fungal richness was governed by host-associated factors such as soil nutrients^38,49^. Our results indicate that the soil TOC, species cover degree, and elevation indirectly and significantly impact the microbial community structure. Regarding the effect of pH, it had the strongest impact on the microbial community, acting significantly, albeit indirectly. This is in agreement with previous studies in which soil pH did not alter the microbial community itself, but directly or indirectly interacted with other soil parameters^36,63^.

Soil properties (TOC and pH) have the greatest impact on the abundance of OTU, which directly affects the microbial community structure. Overall, soil variables often indirectly affect microbial diversity/community. Although rhizosphere soil fungi have a strong and direct impact on microbial community structure, the impact is controlled by host species and soil properties. The overall effect of plant species coverage on microbial diversity/community was significant, indicating that plant species can shape rhizosphere microenvironment. In addition to microbial factors, altitude, vegetation and soil properties affect each other. The altitude and species coverage have the greatest influence on soil pH. Root exudates mainly include mucilage, ecotoenzymes, organic substances, sugars, and various kinds of amino acids. They can significantly improve soil structure, cause soil mineral weathering, increase soil cation exchange capacity, and affect soil pH value, soil surface adsorption characteristics and soil biological characteristics^72^. Therefore, different plant communities have different effects on pH value of rhizosphere soil^73^.

Although vegetation has a strong impact on microbial community structure, altitude as an abiotic factor has the greatest impact on microbial community structure. This is contrary to our initial hypothesis that species may be the most important factor affecting the microbial community structure of rhizosphere soil. The altitude pattern of plant species in Changbai Mountain has been reported previously^33^. With the increase of altitude, the environment becomes harsher and harsher, which, among other things, results in nutritional stress, which leads to great changes in plant community structure^74^. As pH value, soil nutrients and vegetation are widely considered as the main driving factors of soil bacteria. The results showed that the elevation was the main factor affecting the microorganism, and the distribution of plant species, soil organic carbon and pH value was driven indirectly.

## Conclusion

Our results revealed the relationship between vegetation types, elevation, soil properties and soil microbial communities on Changbai Mountain, and found that elevation was the main controlling factor. To the best of our knowledge, this is the first study to reveal that elevation is the main predictor of rhizosphere microbial community structure rather than vegetation in the alpine tundra. It should provide a basis for understanding and predicting species succession and stability of alpine tundra ecosystem.

## Material and methods

### Study area

We performed our sampling in the Changbai Mountains National Nature Reserve (41° 41′ 49″–42° 25′ 18″ N, 127° 42′ 55″–128° 16′ 48″ E). Precisely, the study area is on the north slope of the alpine tundra (42° 01′–42° 05′ N, 128° 03′–128° 07′ E) at an elevation between 2,000 and 2,600 m. Such area is characterized by short growing season (June–September), low temperature (3 °C–7 °C in the growing season) and heavy precipitation (exceeds 1,400 mm annually)^75^. Common plants are dominated by shrubs such as *D. octopetala* L. *var. asiatica*, *Rhododendron aureum Georgi*, *Vaccinium uliginosum Linn*, and tussocks such as *Trollius chinensis, Sanguisorba tenuifolia var. alba*, *Rhodiola cretinii* (*Hamet*) *H. Ohba subsp. sino-alpina* (*Frod*.) *H. Ohba, Ligularia jamesii* (*Hemsl.*) *Kom,* etc^76,77^. The soil is mountain tundra soil, the development of which is very low. The soil layer is thin, and not connected into pieces. Soil profile: 1–5 cm is peat layer, 5–10 cm is peat gravel layer, and 10–30 cm is volcanic rock layer.

### Experimental design

The experimental design was set up two research groups. The first group was used to analyze the effects of altitude and vegetation types on soil microbial community. We set 7 altitudes and at each altitude we selected 3 dominant species communities as research object. So we selected 21 sample locations in all. The coverage of each dominant species in the community was higher than 85%. We selected *R. aureum Georgi* communities at each altitude, because it is the only woody plant with a distribution at each elevation. The other two dominant species plant communities are different at the seven elevations. Detailed distributions can be seen in Supplementary Table S7.

The second research group was used to analyze the direct or indirect effects of altitude, species coverage, and soil physicochemical characteristics on soil microbial community. We set three treatments: The independent community of *R. aureum Georgi* (Da), *R. aureum Georgi* lives with other shrub (Da + S) and *R. aureum Georgi* lives with herbaceous (Da + H). Da + S sites had less than 5% coverage of herbaceous plants, and Da + H sites less than 5% coverage of other shrub. In the three treatments, *R. aureum Georgi* had different cover degree from 40 to 95%, the detailed see Supplementary Table S8. We selected 8 locations of Da + S, 4 locations of Da + H, and the sits of Da in the first and second group are the same locations. Detailed distributions can also be seen in Supplementary Table S8. Therefore, the total sample location number of this research is 33.

### Soil sampling

Soil samples were collected in July of 2014. We randomly selected three 1 × 1 m plots in each location and extracted approximately 500 g soil. As a result, the study included 99 plots. In each plot, we removed the litter layer of the soil and collected plant affected soil samples with five replicates (5 cm diameter × 10 cm deep). As most the plants in the sampling areas grew at relatively high densities (especially for *Rhododendron aureum Georgi*, *Sanguisorba tenuifolia var*, *Ligularia jamesii*), the collected soil was originally only a few millimeters away from the root axis surface, hence they seem to have great impacts on the surrounding soil. Therefore, the collected soil samples were defined as “rhizosphere soil samples”^78^. Except for the 16rDNA sequencing of microbial communities, all the results in this paper are based on the analysis of those 99 plots. By mixing all samples collected from the same location, we sequenced 33 composite soil samples. The collected samples were refrigerated at 4 °C immediately and have been transported to the laboratory within 4 h. They were further sieved by 2 mm mesh sieve and have been assigned into two groups. Half of the samples were stored at 4 °C for chemical analysis and the other half at − 80 °C for microbial community analyses.

### Analysis of soil characteristics and fungal biomass

Soil moisture was measured by the gravimetrical mass loss after drying samples to a constant weight (105 °C for at least 12 h). We measured pH by using a pH meter on a 1:10 (w/v) ratio in distilled water. Soil organic matter was determined by dichromate oxidation with external heat and titration with ferrous ammonium sulphate^79^. TN was determined by semimicro-Kjeldahl (KDY-9820) digestion^80^. Soil total phosphorus (TP) was determined colorimetrically using the molybdate method^81^. MBC and microbial biomass nitrogen (MBN) were determined using the chloroform fumigation extraction method^82^. Soil nitrate nitrogen and ammonium nitrogen were analyzed using a flow-injection autoanalyzer (SKALAR SAN+ +, Netherlands). Phosphatase activity was measured using the modified method of Schinner and von Mersi^83^. Soil catalase activity was measured using the 0.1 N KMnO_4_ titration method^84^. Urease activity was determined according to Klose and Tabatabai^85^. Invertase activity was assayed using the 3,5-dinitrosalicylic acid technique^86^.

Fungal biomass was determined by HPLC analyzing ergosterol content in the soil^87^. Briefly, ergosterol was extracted by KOH, methanol, and ethanol from 3 g of fresh soil (70 °C water bath for 30 min). After n-hexane extraction, concentration, and solvent replacement, ergosterol content was quantified by HPLC (AGILENT 1,200, USA; reverse C18 column; mobile phase, 95% methanol; flow rate, 1 min/ml; detection wavelength, 282 nm).

### Real-time PCR

In order to eliminate the internal variability within each site, we performed 10 replications in the mixed soil samples. Each DNA sample was extracted using 0.3 g freeze-dried soil with the Power Soil DNA Isolation Kit (MOBIO), according to the manufacturer’s protocol. Due to the qPCR is extremely sensitive and vulnerable to interference by humic material. Extracted DNA was purified using the GV-High-Efficiency Agarose Gel DNA Purification Kit (BEI JING DINGGUO CHANGSHENG BIOTECHNOLOGY CO.LTD., China), DNA concentrations were determined using the Qubit quantification platform with QUANI-IT ESDNA BR Assay Kit (INVITROGEN). DNA was diluted to 10 ng/μl and stored at − 80 °C prior to molecular analysis.

The abundances of genes (*cbbl, nifH*, archaeal *amoA*, bacterial *amoA*, *nosZ*) encoding the key enzymes for biological C- and N-cycling were quantified by real-time PCR (IBA7500) for all soil samples. The primers N-cycling functional genes were reference by Mao et al. and Levy-Booth et al.^34,88^, and C-cycling functional genes were reference by Yuan et al.^89^ (details see Supplementary Table S9). Standards for the qPCR assays were generated by PCR products from a common DNA mixture (equal amounts of DNA from all samples)^90^. PCR product was diluted to 100 ng/μl and set five concentration gradients in 10 times diluted. For each sample-derived standard, copy number concentration was calculated based on the length of the PCR product and the mass concentration (measured by Qubit). The 20 μl reaction mixture contained 0.2 μl of each primer (20 μM), 10 μl of 2 × Trans Start Green qPCR Super Mix (TRANSGEN BIOTECH, China), 0.4 μl Passive Reference Dye (50 ×), 0.3 μl of BSA (10 mg/ml), and 10 ng of DNA template.

### Bioinformatics analysis

The 16SrDNA high-throughput sequencing was performed by the REALBIO Genomics Institute (Shanghai, China) using the ILLUMINA MiSeq platform. The 16S V3–V4 region was amplified using the primers U341F (ACT CCT ACG GGA GGC AGC AG) and U806R (GGA CTA CHV GGG TWT CTA AT). The raw data were then subjected to a quality control procedure using UPARSE^91^. The qualified reads were clustered to generate OTUs at the 97% similarity level using USEARCH^92^. A representative sequence for each OTU was assigned to a taxonomic level in the RDP database by the RDP classifier^93^.

A total of 1.8 million high-quality 16S rRNA gene sequence reads were obtained from 33 samples. There were a total of 29,607 distinct OTUs (operational taxonomic units) across samples, with a total of 87,156 unique reads that were assigned to these OTUs. In QIIME, sequences were subsampled to an even depth of 20,067 reads prior to estimating relative taxon abundances and running diversity analyses. Alpha diversity was measured using Chao1, observed species, Shannon, Simpson, goods coverage, and PD whole tree.

### Statistical analysis

All analyses were performed in the corrplot package in R^94^. For soil parameters, one-way analysis of variance (ANOVA) was used to determine differences among elevation treatments and different vegetation treatments. Two-way analysis of variance (ANOVA) was used to the determine differences among elevation × vegetation treatments. The correlations between the abundance of N-cycling functional genes, diversity metrics, and OTU richness and fungal biomass were correlated with soil characteristics using Pearson correlations. Statistical significance was set at *P* < 0.05.

NMDS was performed via the vegan package of R v.3.1.1 project^95^ (Weighted Unifrac scores). We also used analysis of similarities (ANOSIM, 999 permutations) to evaluate the null hypothesis. Heatmaps and clustering tree were constructed using ggplot2 package in R. RDA was performed to determine the environmental factors that significantly correlated with community composition (abundance of OTUs), via the function envfit (999 permutations) in the vegan package of R v.3.1.1 project^96,97^. SEM was constructed via AMOS 17.0^98^ and the ‘vegan’ package in R. Only abiotic factors variables that were both significant correlated (P < 0.05) with OTU richness, fungal biomass, elevation and vegetation coverage were included in the model construction, namely soil TOC and pH. We tested the fitness of the model with the data using the maximum likelihood (λ^2^) goodness-of-fit test, Jöreskog’s GFI, the Bollen–Stine bootstrap test and RMSEA.

## Supplementary information

Supplementary Information
