# Supplementary material for: Elevational is the main factor controlling the soil microbial community structure in alpine tundra of the Changbai Mountain
Source: Sci Rep. 2020 Jul 24;10:12442. doi: 10.1038/s41598-020-69441-w (PMC7381615; doi:10.1038/s41598-020-69441-w)
Supplement: Supplementary file 1 — Supplementary Information [file 41598_2020_69441_MOESM1_ESM.docx]

**Elevational is the main factor controlling the soil microbial community structure in alpine tundra of the Changbai Mountain**

Mingze Tang ^1^, Lin Li ^1^, Xiaolong Wang ^1^, Jian You ^1^, Jiangnan Li ^1^, Xia Chen ^1*^

1.National & Local United Engineering Laboratory for Chinese Herbal Medicine Breeding and Cultivation, School of Life Sciences, Jilin University, Changchun 130112, China.

^*^For correspondence. E-mail address: [chenxiajlu@163.com](mailto:chenxiajlu@163.com)

TableS1. Soil variables (means ± standard deviation) were from seven elevations. Each elevation has three sampling points. The lower case letters ‘a’, ‘b’, ‘c’ and ‘d’ indicate contrasts that are significantly different (P < 0.05) between different treatment.

|  | | TN | TOC | TP | NO_3_^-^ | NH_4_^+^ | MBC | MBN | Moisture | pH | CN | Sucrase | Phosphatase | Catalase | Urease | |
| --- | --- | --- | --- | --- | --- | --- | --- | --- | --- | --- | --- | --- | --- | --- | --- | --- |
| 2600 | Da | 6.4±0.03a | 106.3±0.63c | 410.6±6.10a | 93.6±0.79a | 141.2±1.39a | 1027.1±31.80a | 241.6±22.22a | 17.9±0.50b | 5.4±0.02c | 16.6±0.13a | 30.9±2.45a | 9.5±0.09a | 35.0±1.33a | 34.2±0.92a |  |
|  | Do | 2.2±0.10c | 20.8±1.06b | 284.0±3.69c | 52.5±0.54c | 115.6±2.26b | 621.7±53.34b | 154.4±7.37b | 24.1±1.01a | 5.6±0.01b | 9.5±0.89b | 11.3±0.33b | 3.8±0.09b | 20.3±0.77b | 7.9±0.41b |  |
|  | Sr | 2.8±0.04b | 29.1±0.57a | 348.6±2.04b | 70.7±0.61b | 113.1±0.61b | 705.2±51.97b | 187.1±22.52b | 23.4±1.36a | 5.6±0.02a | 10.3±0.23b | 10.0±0.66b | 3.9±0.10b | 20.0±0.76b | 8.6±0.82b |  |
| 2500 | Da | 8.3±0.05a | 148.8±1.90b | 840.2±7.35a | 39.7±0.50a | 260.7±1.08a | 1588.5±63.05a | 476.3±11.75a | 37.9±1.67b | 5.1±0.01b | 18.0±0.17a | 36.6±1.89a | 9.8±0.09a | 33.4±1.27a | 33.6±0.90b |  |
|  | Pc | 6.9±0.16b | 106.7±1.57a | 698.9±3.56c | 29.6±2.49b | 197.3±1.55b | 1283.2±32.49b | 415.3±26.20b | 42.0±0.82a | 5.1±0.02b | 15.5±0.15b | 25.6±1.77b | 10.0±0.10b | 32.8±1.24a | 17.5±1.12a |  |
|  | Sr | 6.6±0.26b | 105.1±1.22b | 454.1±1.50b | 13.5±0.34c | 198.7±1.96b | 865.2±63.43c | 223.2±26.77c | 42.0±0.64a | 5.0±0.2a | 16.0±0.82b | 25.7±1.38b | 9.9±0.10ab | 34.0±1.29a | 39.5±2.10c |  |
| 2400 | Da | 5.9±0.15b | 118.2±1.68a | 492.7±0.70c | 19.1±0.61c | 91.3±2.32a | 1439.6±31.72a | 190.1±1.99b | 49.2±0.57a | 4.9±0.01a | 20.1±0.23a | 20.0±0.91b | 9.9±0.10a | 33.9±1.29a | 29.3±7.01a |  |
|  | Do | 5.1±0.03a | 71.7±0.72b | 186.9±2.03a | 29.4±1.68a | 107.2±0.66b | 1662.2±109.32b | 160.0±16.32c | 43.6±0.79b | 4.8±0.01c | 14.2±0.22b | 24.5±1.90c | 10.0±0.10ab | 34.0±1.39a | 28.0±1.07a |  |
|  | Vs | 5.6±0.07c | 84.3±1.88c | 618.3±6.04b | 26.6±0.82b | 111.5±1.11c | 1548.1±32.54b | 382.6±10.25a | 49.1±0.14a | 4.9±0.01b | 15.0±0.48c | 29.9±1.11a | 10.2±0.10b | 34.4±1.31a | 8.5±0.83b |  |
| 2300 | Da | 7.2±0.27a | 115.4±1.03a | 622.9±3.18a | 23.0±0.34c | 116.9±1.39a | 1408.8±24.04a | 337.1±10.11b | 47.9±0.46b | 5.1±0.01a | 16.1±0.75a | 33.9±0.87b | 10.1±0.10a | 32.7±1.24a | 32.3±1.59a |  |
|  | Ss | 6.5±0.03b | 74.7±0.63c | 574.0±2.00c | 114.5±2.26a | 125.6±1.92b | 1235.0±43.82b | 273.3±33.56c | 36.0±1.06c | 4.8±0.02c | 11.4±0.14b | 20.2±2.67a | 9.9±0.10a | 25.7±0.98b | 22.2±2.38c |  |
|  | Pc | 6.2±0.11c | 101.9±0.80b | 557.6±2.65b | 17.8±0.27b | 117.5±1.46a | 1434.3±55.00a | 389.9±14.92a | 67.7±0.71a | 5.0±0.03b | 16.5±0.20a | 18.0±1.66a | 14.4±0.43b | 34.3±1.30a | 39.7±4.59b |  |
| 2200 | Da | 8.9±0.50a | 222.5±2.09a | 476.4±3.79a | 15.4±0.39c | 68.7±1.34b | 1898.2±64.20a | 382.2±24.49a | 67.5±0.44a | 4.6±0.01b | 25.2±1.57a | 35.6±2.14b | 7.2±0.23a | 27.7±1.05a | 56.4±2.25b |  |
|  | Vs | 7.5±0.14b | 124.8±0.44c | 327.2±1.17b | 29.6±0.94a | 170.8±2.61a | 1621.7±53.34b | 303.6±27.98c | 20.7±1.18c | 5.5±0.03a | 16.7±0.27c | 42.3±1.90c | 7.3±0.24a | 34.9±1.33b | 44.9±1.70a |  |
|  | Rc | 7.6±0.27b | 169.7±1.85b | 314.4±2.82c | 25.8±0.44b | 114.0±1.58c | 1610.0±31.66b | 237.4±25.24b | 58.2±0.39b | 5.0±0.01c | 22.4±0.92b | 30.9±1.18a | 7.1±0.23a | 23.9±0.91c | 50.2±1.56c |  |
| 2100 | Da | 6.7±0.12b | 119.7±0.79c | 353.8±1.16c | 21.4±1.10a | 78.1±1.70c | 1017.7±74.89a | 403.3±33.85a | 68.7±1.37a | 4.9±0.02b | 17.9±0.20c | 26.1±0.79b | 7.1±0.23a | 35.4±1.34a | 52.3±1.57b |  |
|  | Vs | 7.2±0.03a | 215.7±2.08a | 360.9±2.52b | 19.1±0.13b | 48.3±1.26b | 1219.8±41.35b | 328.5±32.90b | 68.1±0.54a | 4.8±0.01c | 29.9±0.41a | 22.9±1.39a | 6.3±0.21b | 33.2±1.26ab | 63.7±2.11a |  |
|  | Do | 7.3±0.17a | 155.1±1.03b | 495.1±0.47a | 28.5±0.74b | 142.2±3.59a | 1724.8±32.05c | 255.6±13.69c | 47.0±0.49b | 5.0±0.01a | 21.3±0.16b | 26.8±1.96b | 7.0±0.23a | 30.8±1.17b | 28.2±0.86c |  |
| 2000 | Da | 7.6±0.06ab | 109.0±1.57a | 307.4±2.46b | 23.6±2.21a | 171.6±2.66b | 1308.7±41.14b | 337.6±40.38c | 43.7±0.57a | 5.0±0.03b | 14.4±0.62 | 25.5±0.90b | 5.9±0.19b | 34.9±1.32a | 18.2±1.08b |  |
|  | Vs | 7.3±0.17a | 93.5±1.65b | 94.0±1.34a | 19.2±1.11bc | 130.6±2.80a | 1056.2±95.19a | 235.5±19.28b | 41.7±0.94b | 5.4±0.02a | 12.8±0.34b | 26.0±1.58b | 5.8±0.19b | 34.7±1.32a | 35.8±2.85a |  |
|  | Do | 6.7±0.35c | 137.7±1.17c | 315.1±4.39c | 22.6±1.07c | 77.3±2.42c | 1499.0±75.06c | 377.2±35.45a | 43.8±0.60b | 4.9±0.02c | 20.7±0.97a | 21.5±0.55a | 7.4±0.24a | 28.7±1.09b | 56.1±1.71c |  |
| Elevation（E） | | ** | * | * | – | * | * | – | * | ** | – | – | – | – | ** |  |
| Plant species（P） | | *** | *** | *** | *** | *** | *** | *** | *** | *** | *** | *** | *** | *** | *** |  |
| E*P | | *** | *** | *** | *** | *** | *** | *** | *** | *** | *** | *** | *** | *** | *** |  |

TableS2. Relationships between C- and N-cycling and soil properties. Green represents a significant (*P*<0.05) positive correlation, Yellow represents a significant (*P*<0.05) negative correlation. The value is correlations (*r*).

|  | bacterial *AMO* | archaeal *AMO* | *nifH* | *nosZ* | *cbbl* |
| --- | --- | --- | --- | --- | --- |
| TN | － | － | － | － | － |
| TOC | － | － | － | － | 0.526 |
| TP | － | － | － | － | － |
| NO_3_^-^ | － | － | － | － | － |
| NH_4_^+^ | － | － | － | － | -0.479 |
| MBC | － | － | － | － | － |
| MBN | － | － | － | － | － |
| Moisture | 0.654 | -0.465 | － | － | 0.485 |
| pH | -0.441 | － | 0.453 | -0.557 | -0.679 |
| CN | － | － | － | － | 0.561 |
| Urease | － | -0.463 | － | － | 0.598 |
| Sucrase | － | － | － | － | － |
| Catalase | － | － | － | － | － |
| Acid Phosphatase | － | － | － | 0.550 | － |
| elevation | － | 0.710 | － | － | － |

TableS3. Relative abundances of the dominant bacterial phyla in soils separated according to elevation categories. There were significant differences between communities at different elevations. Bolded values indicate significant (*P*<0.05) effects.

| Phylum | 2600 | 2500 | 2400 | 2300 | 2200 | 2100 | 2000 | P-value |
| --- | --- | --- | --- | --- | --- | --- | --- | --- |
| Acidobacteria | 0.311 | 0.39 | 0.242 | 0.314 | 0.316 | 0.3 | 0.417 | 0.141 |
| Proteobacteria | 0.311 | 0.23 | 0.197 | 0.287 | 0.324 | 0.318 | 0.268 | 0.185 |
| Actinobacteria | 0.127 | 0.12 | 0.16 | 0.225 | 0.208 | 0.206 | 0.129 | 0.118 |
| Chloroflexi | 0.019 | 0.03 | 0.071 | 0.008 | 0.011 | 0.02 | 0.05 | 0.079 |
| Bacteroidetes | 0.039 | 0.01 | 0.009 | 0.013 | 0.016 | 0.015 | 0.011 | **0.005** |
| Candidatus Saccharibacteria | 0.012 | 0.01 | 0.013 | 0.005 | 0.012 | 0.013 | 0.007 | 0.443 |
| Gemmatimonadetes | 0.018 | 0 | 0.003 | 0.007 | 0.01 | 0.005 | 0.006 | **0.002** |
| Planctomycetes | 0.001 | 0.01 | 0.01 | 0.006 | 0.006 | 0.009 | 0.008 | 0.469 |
| Firmicutes | 0.004 | 0 | 0.004 | 0.008 | 0.002 | 0.004 | 0.004 | 0.511 |
| Verrucomicrobia | 0.001 | 0 | 0.001 | 0.002 | 0.001 | 0.002 | 0.003 | 0.468 |
| Cyanobacteria/Chloroplast | 0.003 | 0 | 0.001 | 0.001 | 0.001 | 0.001 | 0.002 | 0.074 |
| Latescibacteria | 0.002 | 0 | 0 | 0 | 0.001 | 0 | 0.004 | 0.143 |
| Nitrospirae | 0.002 | 0 | 0 | 0.001 | 0 | 0 | 0 | **0.002** |
| Deinococcus-Thermus | 0 | 0 | 0 | 0.001 | 0 | 0 | 0 | 0.553 |

TableS4. Soil variables (means ± standard deviation) were from three treatments. The lower case letters ‘a’, ‘b’, ‘c’ and ‘d’ indicate contrasts that are significantly different (P < 0.05) between different treatment.

|  | Da | Da+S | Da+H |
| --- | --- | --- | --- |
| TN | 7.35±1.07a | 7.39±1.19a | 6.55±0.77a |
| TOC | 146.63±40.81a | 124.59±38.71a | 95.65±22.56b |
| TP | 546.38±168.56a | 453.24±250.33a | 614.31±125.52a |
| NO_3_^-^ | 24.42±8.06a | 25.41±2.66a | 24.86±5.89a |
| NH_4_^+^ | 126.31±71.08a | 104.56±23.21a | 136.47±23.30a |
| MBC | 1512.92±303.46a | 1472.27±283.45a | 1219.62±171.1a1 |
| MBN | 340.76±103.89a | 332.21±95.76a | 367.97±41.21a |
| Moisture | 53.01±12.34a | 49.66±7.96a | 51.49±9.74a |
| pH | 4.93±0.18a | 5.01±0.30ab | 5.13±0.28b |
| CN | 19.78±3.22b | 16.87±5.69ab | 14.43±2.26a |
| Urease | 37.01±14.55a | 34.02±14.67a | 29.78±15.90a |
| Sucrase | 29.63±6.71a | 31.28±8.89a | 25.48±7.69a |
| Catalase | 32.98±2.78a | 29.36±5.64a | 31.06±4.26a |
| Phosphatase | 8.34±1.81b | 8.73±2.44b | 12.44±2.40a |

|  | TOC | pH | Elevation | Fungal biomass | OTU richness | Cover degree |
| --- | --- | --- | --- | --- | --- | --- |
| TOC | 1 |  |  |  |  |  |
| pH | -0.616** | 1 |  |  |  |  |
| Elevation | -0.477* | 0.624** | 1 |  |  |  |
| Fungal biomass | 0.774** | -0.476* | -0.497* | 1 |  |  |
| OTU richness | 0.679** | -0.717** | -0.352 | 0.563** | 1 |  |
| Cover degree | 0.625** | -0.605** | -0.303 | 0.721** | 0.620** | 1 |

TableS5. Pearson correlation coefficient (*r*) between soil properties elevation, OTU richness and plant cover degree, Asterisk indicates significant differences *indicate significant (*P*<0.05) effects, ** indicate significant (*P*<0.01) effects.

TableS6. The correlation between community composition and environmental variables for bacteria. Values in bold indicate significant correlation (*P*<0.05).

|  | *r2* | *Pr (>r)* |
| --- | --- | --- |
| Fungal biomass | 0.62 | **0.002** |
| Cover degree | 0.37 | **0.032** |
| Moisture | 0.3 | **0.05** |
| pH | 0.36 | 0.06 |
| Elevation | 0.28 | 0.07 |
| TOC | 0.29 | 0.08 |
| NO_3_^-^ | 0.19 | 0.20 |
| C/N | 0.13 | 0.35 |
| TN | 0.1 | 0.46 |
| NH_4_^+^ | 0.06 | 0.57 |
| MBC | 0.05 | 0.72 |
| MBN | 0.05 | 0.72 |
| TK | 0.04 | 0.77 |
| TP | 0.03 | 0.81 |

Table S7 Sampling sites, geographical location and dominant species that use plant coverage (%) to indicate.

| **Altitude(m)** | **Types** | **Latitude** | **Longitude** | **Dominant species & Coverage (%)** |
| --- | --- | --- | --- | --- |
| 2600 | Ra | 42°02′92″ | 128°06′64″ | *Rhododendron aureum Georgi* 92% |
| 2600 | Do | 42°02′92″ | 128°06′64″ | *Dryas octopetala L.* 95% |
| 2600 | Sr | 42°02′92″ | 128°06′64″ | *Salix rotundifolia* 97% |
| 2500 | Ra | 42°01′944″ | 128°04′009″ | *Rhododendron aureum Georgi* 90% |
| 2500 | Pc | 42°01′944″ | 128°04′009″ | *Phyllodoce caerulea (L.) Bab.* 90% |
| 2500 | Sr | 42°01′944″ | 128°04′009″ | *Salix rotundifolia 94%* |
| 2400 | Ra | 42°02′132″ | 128°03′979″ | *Rhododendron aureum Georgi* 89% |
| 2400 | Vs | 42°02′132″ | 128°03′979″ | *Vaccinium uliginosum Linn.* 94% |
| 2400 | Do | 42°02′132″ | 128°03′979″ | *Dryas octopetala L.* 93% |
| 2300 | Ra | 42°04′018″ | 128°06′78″ | *Rhododendron aureum Georgi* 87% |
| 2300 | Vs | 42°04′018″ | 128°06′78″ | *Vaccinium uliginosum Linn.* 92% |
| 2300 | Pc | 42°04′018″ | 128°06′78″ | *Phyllodoce caerulea (L.) Bab.* 91% |
| 2200 | Ra | 42°04′202″ | 128°06′86″ | *Rhododendron aureum Georgi* 95% |
| 2200 | Vs | 42°04′202″ | 128°06′86″ | *Vaccinium uliginosum Linn.* 90% |
| 2200 | Rc | 42°04′202″ | 128°06′86″ | *Rhododendron confertissimum Nakai* 95% |
| 2100 | Ra | 42°04′575″ | 128°07′06″ | *Rhododendron aureum Georgi* 85% |
| 2100 | Vs | 42°04′575″ | 128°07′06″ | *Vaccinium uliginosum Linn.* 92% |
| 2100 | Do | 42°04′575″ | 128°07′06″ | *Dryas octopetala L.* 96% |
| 2000 | Ra | 42°05′552″ | 128°07′99″ | *Rhododendron aureum Georgi* 95% |
| 2000 | Vs | 42°05′552″ | 128°07′99″ | *Vaccinium uliginosum Linn.* 94% |
| 2000 | Do | 42°05′552″ | 128°07′99″ | *Dryas octopetala L.* 94% |

TableS8. Sampling sites, geographical location and dominant species that use plant coverage (%) to indicate.

| **Types** | **Altitude(m)** | **Latitude** | **Longitude** | **Dominant species & Coverage (%)** |
| --- | --- | --- | --- | --- |
| Da | 2500 | 42°01′944″ | 128°04′009″ | Rhododendron aureum Georgi 90% |
| Da+S | 2500 | 42°01′944″ | 128°04′009″ | Rhododendron aureum Georgi 40% Phyllodoce caerulea (L.) Bab. 52% |
| Da | 2400 | 42°02′132″ | 128°03′979″ | Rhododendron aureum Georgi 89% |
| Da+S | 2400 | 42°02′132″ | 128°03′979″ | Rhododendron aureum Georgi 52% Phyllodoce caerulea (L.) Bab. 44% |
| Da+S | 2400 | 42°02′132″ | 128°03′979″ | Rhododendron aureum Georgi 65% Vaccinium uliginosum Linn. 19% Dryas octopetala L. 9% |
| Da+H | 2400 | 42°02′132″ | 128°03′979″ | Rhododendron aureum Georgi 40% Sanguisorba sitchensis C. A. Mey. 53% |
| Da | 2300 | 42°04′018″ | 128°06′78″ | Rhododendron aureum Georgi 87% |
| Da+H | 2300 | 42°04′018″ | 128°06′78″ | Rhododendron aureum Georgi 55% Sanguisorba sitchensis C. A. Mey. 43% |
| Da | 2200 | 42°04′202″ | 128°06′86″ | Rhododendron aureum Georgi 95% |
| Da+S | 2200 | 42°04′202″ | 128°06′86″ | *Rhododendron aureum Georgi 50% Vaccinium uliginosum* Linn. 41% |
| Da+S | 2200 | 42°04′202″ | 128°06′86″ | Rhododendron aureum Georgi 65% Rhododendron confertissimum Nakai 20% Vaccinium uliginosum Linn. 11% |
| Da | 2100 | 42°04′575″ | 128°07′06″ | Rhododendron aureum Georgi 85% |
| Da+S | 2100 | 42°04′575″ | 128°07′06″ | *Rhododendron aureum Georgi 75% Vaccinium uliginosum* Linn. 20% |
| Da+H | 2100 | 42°04′575″ | 128°07′06″ | Rhododendron aureum Georgi 75% Sanguisorba sitchensis C. A. Mey. 20% |
| Da | 2000 | 42°05′552″ | 128°07′99″ | Rhododendron aureum Georgi 95% |
| Da+S | 2000 | 42°05′552″ | 128°07′99″ | *Rhododendron aureum Georgi 70% Vaccinium uliginosum* Linn. 14% |
| Da+S | 2000 | 42°05′552″ | 128°07′99″ | Rhododendron aureum Georgi 53% Dryas octopetala L.24% Vaccinium uliginosum Linn. 15% |
| Da+H | 2000 | 42°05′552″ | 128°07′99″ | Rhododendron aureum Georgi 65% Sanguisorba sitchensis C. A. Mey. 32% |

TableS9. Primers and real-time PCR conditions used in this experiment.

| Function genes | Primers | | Real-time PCR protocol |
| --- | --- | --- | --- |
| *nifH* | PolF | 5′-TG CGAY CCS AAR GCB GAC TC-3′ | 94°C for 5 min; 40 cycles of 94°C for 30s, 57°C for 45s(54°C for *nifH* gene, 60°C for bacterial *AMO*), 72°C for 1 min. |
|  | PolR | 5′-ATS GCC ATC ATY TCR CCG GA-3′ |  |
|  |  |  |  |
| archaeal *AMO* | *Arch-amoA*F | 5′-STA ATG GTC TGG CTT AGA CG-3′ |  |
|  | *Arch-amoA*R | 5′-GCG GCC ATC CAT CTG TAT GT-3′ |  |
|  |  |  |  |
| bacterial *AMO* | *amoA*-1F | 5′-GGG GTT TCT ACT GGT GGT-3′ |  |
|  | *amoA*-2R | 5′-CCC CTC KGS AAA GCC TTC TTC-3′ |  |
|  |  |  |  |
| *nosZ* | *nosZ*-F | 5′-CGY TGT TCM TCG ACA GCC AG-3′ |  |
|  | *nosZ*-1622R | 5′-CGS ACC TTS TTG CCS TYG CG-3′ |  |
|  |  |  |  |
| *cbbl* | K2f | 5′-ACCAYCAAGCCSAAGCTSGG-3′ |  |
|  | V2r | 5′-GCCTTCSAGCTTGCCSACCRC-3′ |  |


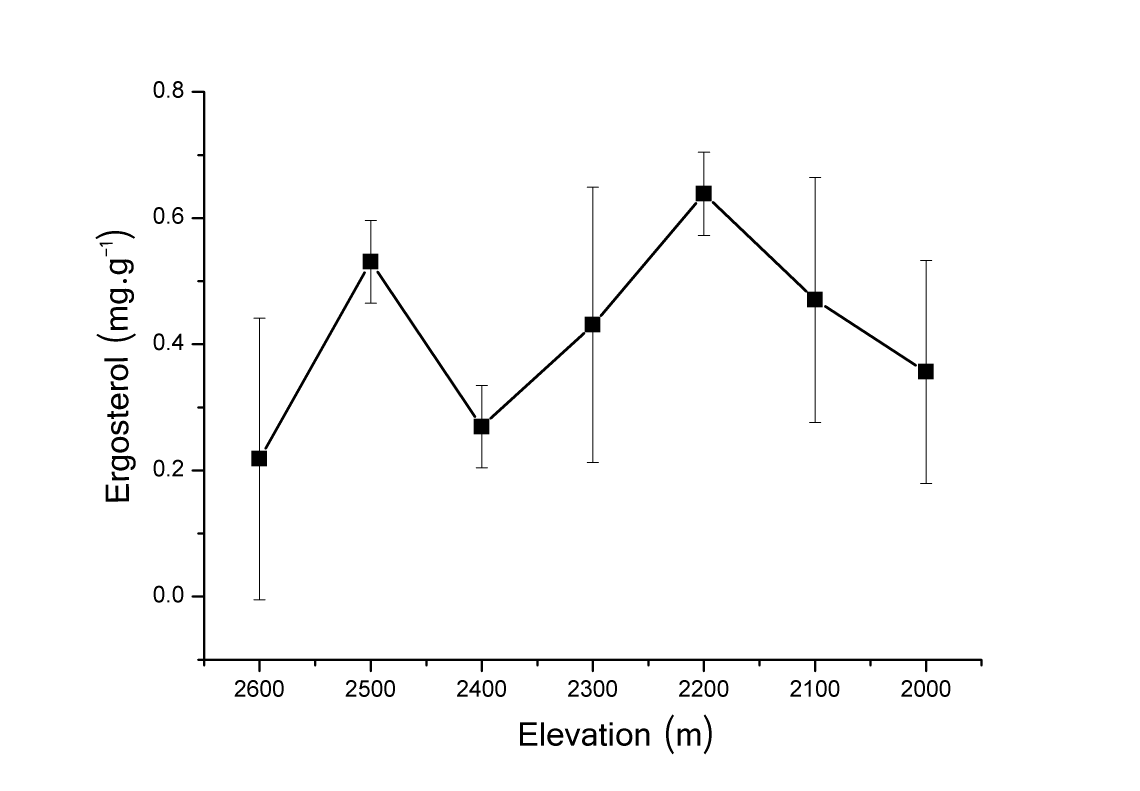


FigS1. Ergosterol (mg.g^-1^) in rhizosphere soil for different elevations.


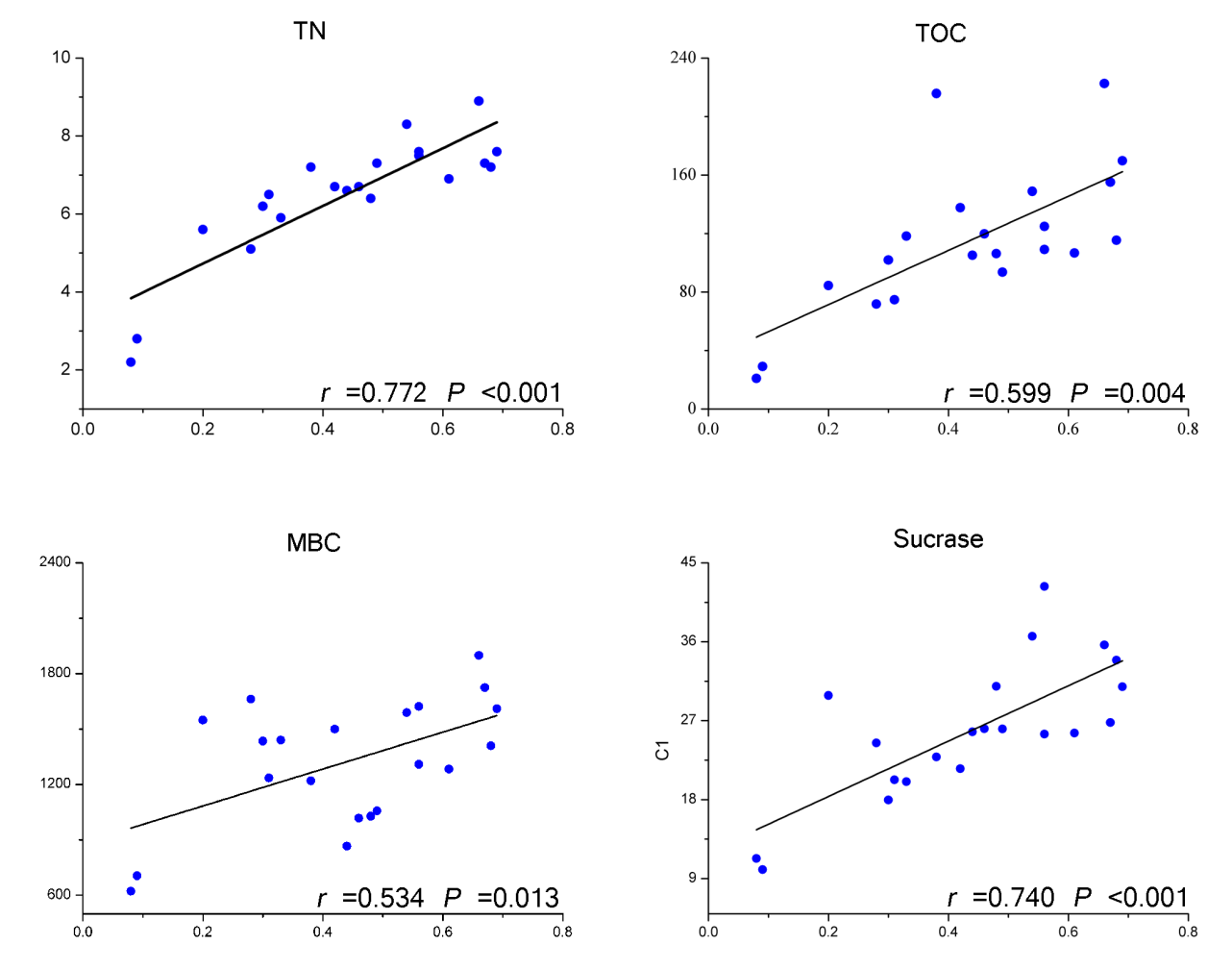


FigS2. Pearson correlations between fungal biomass and soil properties.


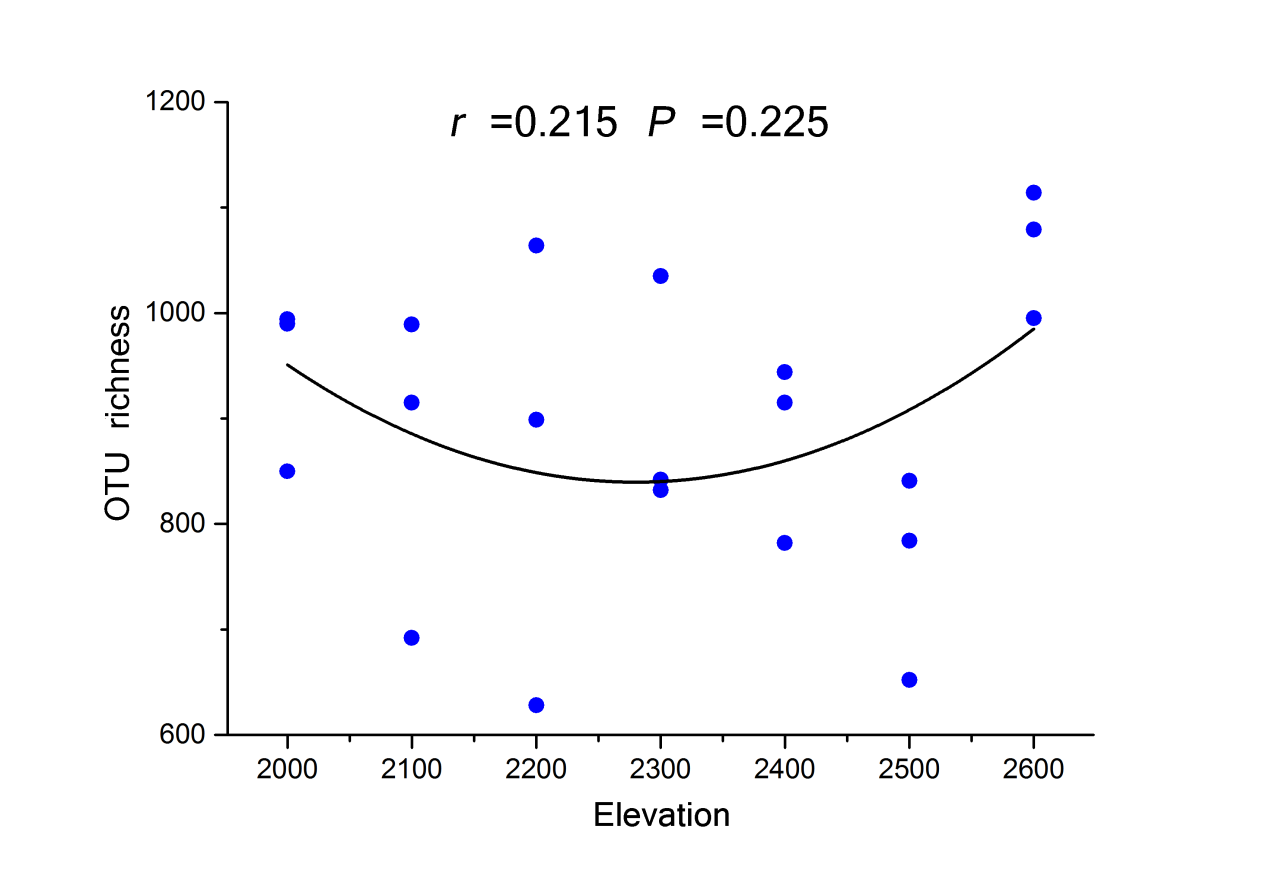


FigS3. Pearson correlations between OTU richness and elevations.


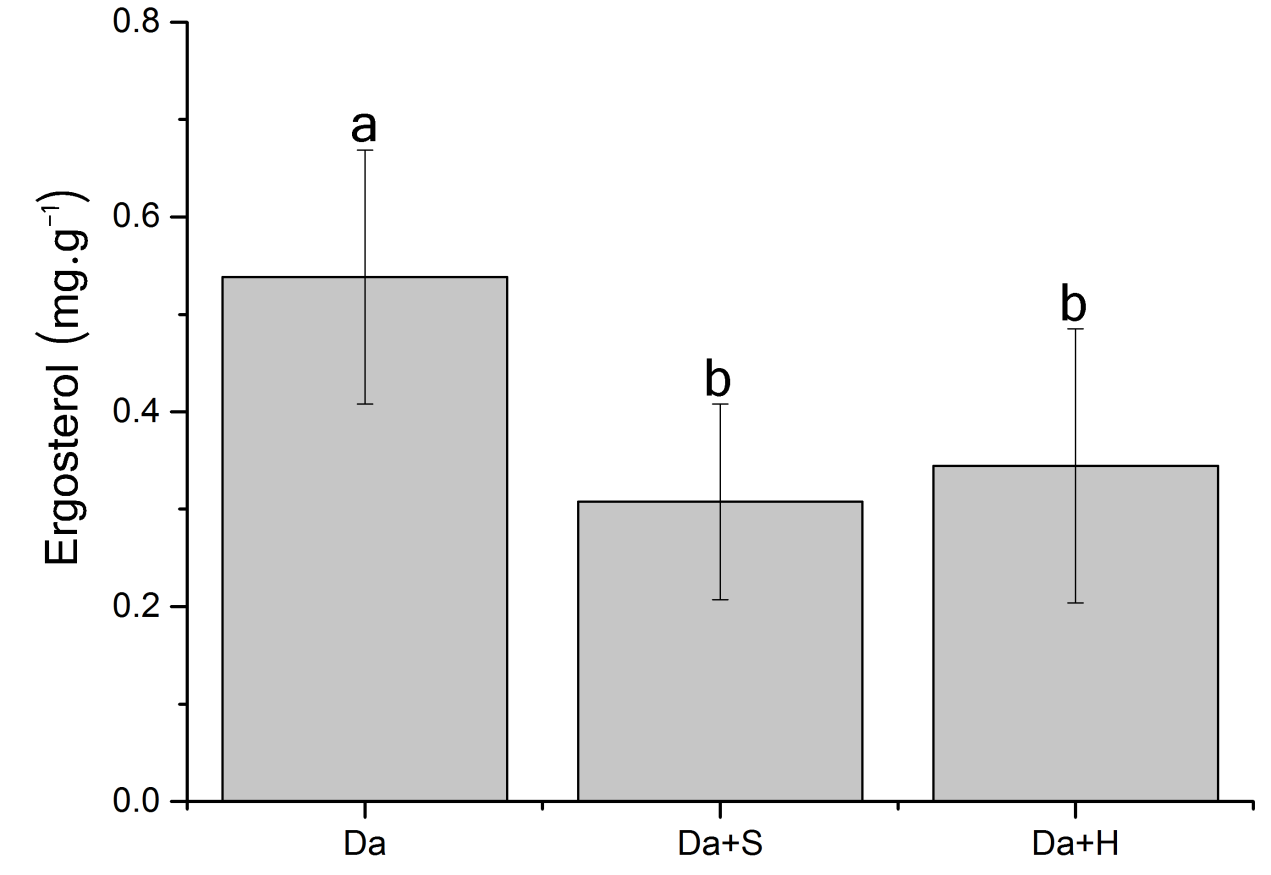


FigS4. Ergosterol (mg.g-1) in rhizosphere soil for Da, Da+S, Da+H treatments.


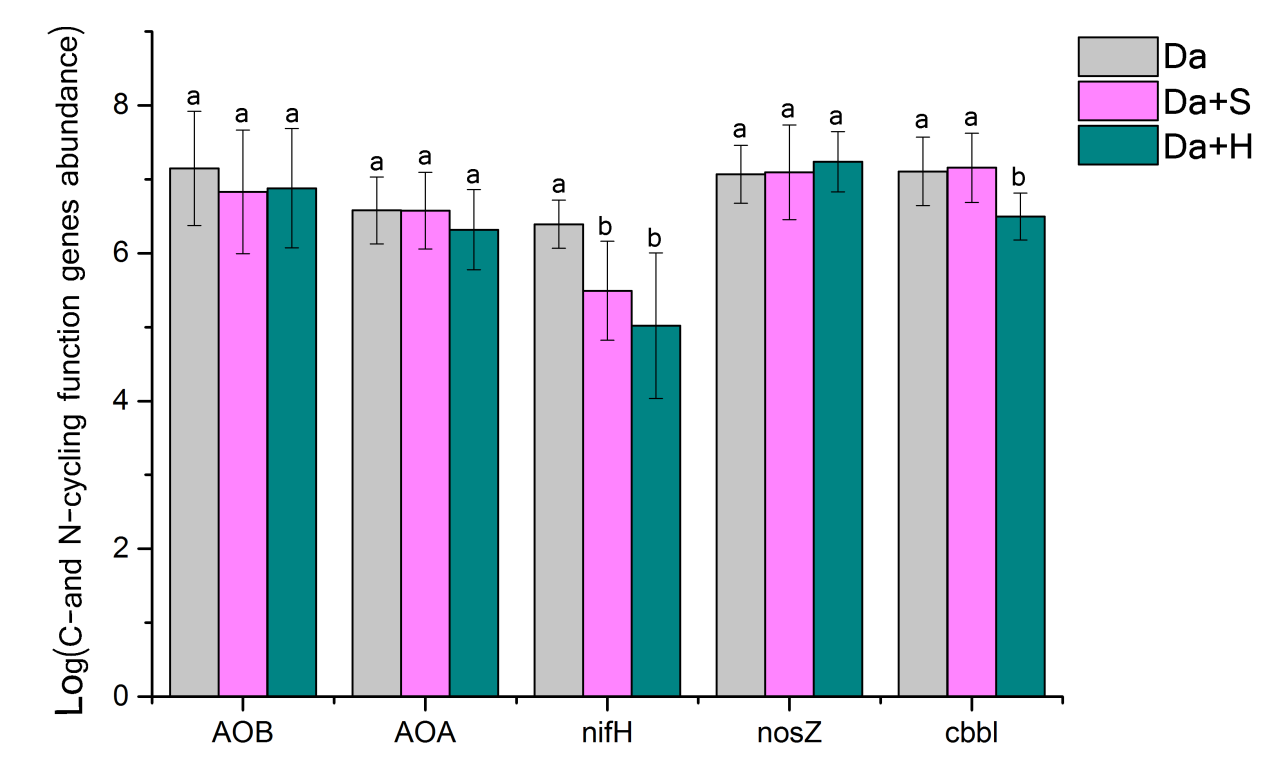


FigS5. Real-time PCR quantification of the C- and N-cycling function genes (*cbbl*, *nifH*, archaeal *AMO*, bacterial *AMO* and *nosZ*) in the rhizosphere soil under three treatments.


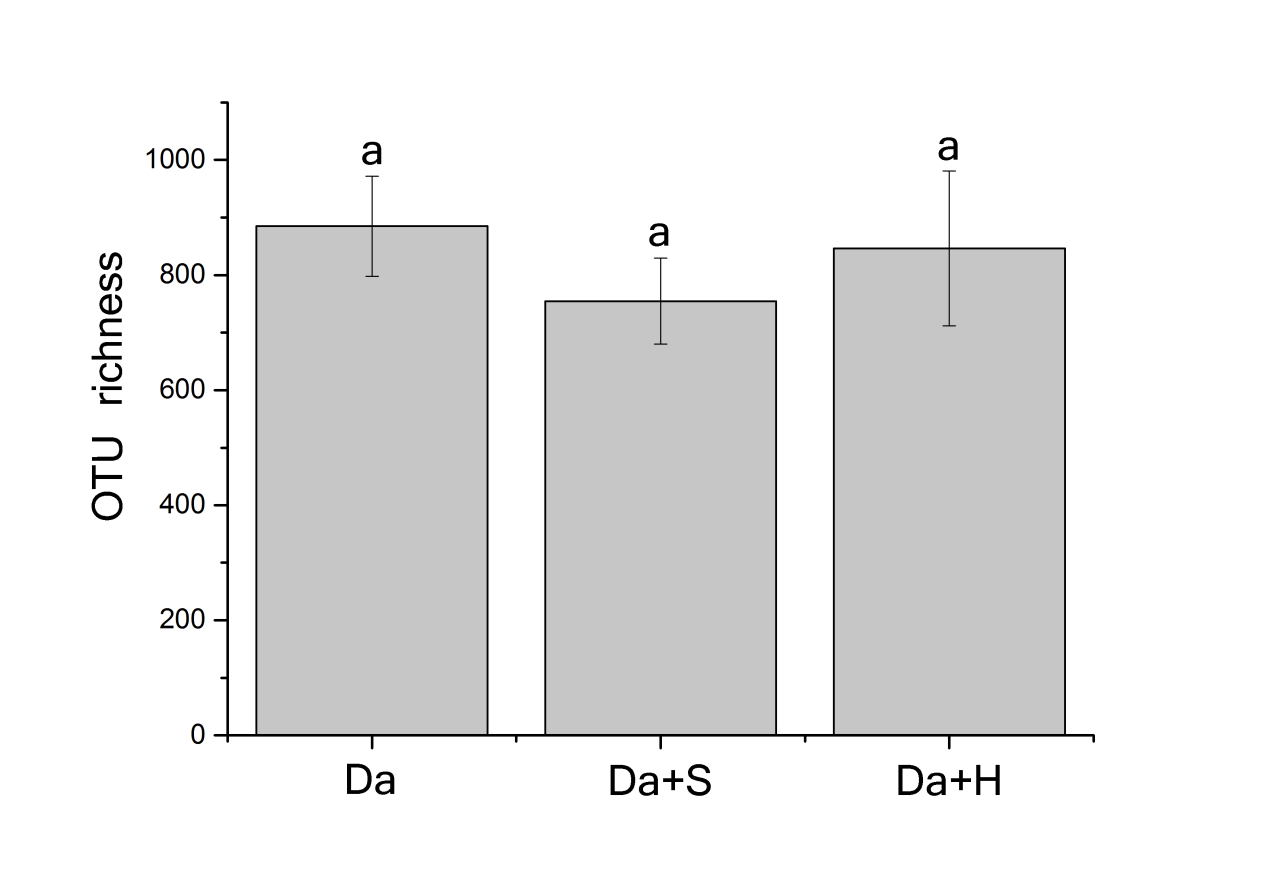


FigS6. The value of OTU richness under three treatments.
